# CRACC-CRACC Interaction between Kupffer and NK Cells Contributes to Poly I:C/D-GalN Induced Hepatitis

**DOI:** 10.1371/journal.pone.0076681

**Published:** 2013-09-30

**Authors:** Yangxi Li, Guoshuai Cao, Xiaodong Zheng, Jun Wang, Haiming Wei, Zhigang Tian, Rui Sun

**Affiliations:** 1 Department of Immunology, School of Life Sciences, University of Science and Technology of China, Hefei, Anhui, China; 2 Hefei National Laboratory for Physical Sciences at Microscale, Hefei, Anhui, China; National Institutes of Health, United States of America

## Abstract

CD2-like receptor activating cytotoxic cells (CRACC) is known as a critical activating receptor of natural killer (NK) cells. We have previously reported that NK cells contribute to Poly I:C/D-galactosamine (D-GalN)-induced fulminant hepatitis. Since natural killer group 2, member D (NKG2D) is considered critical but not the only activating receptor for NK cells, we investigated the role of CRACC in this model. We found that CRACC was abundant on hepatic NK cells but with low expression levels on Kupffer cells under normal conditions. Expression of CRACC on NK cells and Kupffer cells was remarkably upregulated after poly I:C injection. Hepatic CRACC mRNA levels were also upregulated in Poly I:C/D-GalN-treated mice, and correlated positively with the serum alanine aminotransferase (ALT) levels. CRACC expression on Kupffer cells was specifically silenced by nano-particle encapsulated siRNA *in vivo*, which significantly reduced Poly I:C/D-GalN-induced liver injury. In co-culture experiments, it was further verified that silencing CRACC expression or blockade of CRACC activation by mAb reduced the production of interferon (IFN)-γ and tumor necrosis factor (TNF)-α. Collectively, our findings suggest that CRACC-CRACC interaction between NK cells and resident Kupffer cells contributes to Poly I:C/D-GalN-induced fulminant hepatitis.

## Introduction

The liver is not only the largest digestive glands but also the critical portal to the microorganisms derived from digestive tract. Emerging evidence suggests that the liver is considered as an innate immunity associated organ, because liver immune cells are enriched in innate immune cells including NK cells, NKT cells, Kupffer cells and γδT cells [1], compared with peripheral blood and other organs. The immunomodulation among these cells is critical to the orchestration of immune reaction. In many models of liver injury, innate immune cells were found to interact with each other or effect adoptive immune cells to exert immunopathogenic effect [2–7].

Our previous study has established an acute liver injury model induced by poly I:C and D-galactosamine (D-GalN) [6]. In this model, activation of natural killer group 2, member D (NKG2D) by recognizing retinoic acid early inducible-1 (Rae1) on Kupffer cells induces NK cell-mediated fulminant hepatitis. NKG2D-Rae-1interaction is believed a trigger of NK cells activation; however the blockade of NKG2D by a monoclonal antibody only partially prevent the hepatitis, which implied that other activating receptors may also contribute to the interaction between NK cells and Kupffer cells.

The signaling lymphocytes activating molecule (SLAM) family members are surface receptors broadly expressed on hematopoietic cells and orchestrate the cooperation among them [8–11]. And a recent study has demonstrated that tumor derived monocytes are responsible to the impaired functional activities of NK cells by CD48/2B4 interaction [12]. Thus, it is tempting to speculate that SLAM family could participate in the hepatic innate immunomodulation. CD2-like receptor activating cytotoxic cells (CRACC) is a cell surface receptor as a member of the SLAM family. CRACC was reported to be expressed on natural killer cells (NK cells), natural killer T cells (NKT cells), B cells, activated T cells and dendritic cells (DCs) under normal conditions [13–16]. It is commonly considered an activating receptor on NK cells [14,17]. The altered expression of CRACC was observed under a few immunopathogenic conditions, including systemic lupus erythematosus (SLE), rheumatoid arthritis (RA), multiple myeloma (MM) and NK cells mediated aggressive periodontitis [18–21]. And CRACC expression on splenic NK cells has been reported to be upregulated by Poly I:C in vivo [14]. It is reasonable to speculate that CRACC is an activating receptor on NK cells involved in this Poly I:C/D-GalN induced hepatitis model.

RNA interfere (RNAi) is a common method to suppress protein expression at mRNA levels. However, the application of RNAi to immune cells is still limited by the transfection efficiency. It is reported that lipid-based nanoparticle is capable of delivering siRNA to Kupffer cells efficiently [22,23]. It provides us a chance to interfere the protein expression of Kupffer cells by siRNA.

This study is to investigate the role of CRACC-CRACC interaction between Kupffer cells and NK cells in the hepatitis induced by Poly I:C/D-GalN. Briefly, we found Poly I:C stimulation markedly elevated the expression of CRACC on both Kupffer cells and NK cells; and CRACC interaction between NK cells and Kupffer cells contributed to the Poly I:C/D-GalN induced liver injury by increasing the production of IFN-γ and TNF-α.

## Materials and Methods

### Mice and Ethics Statement

Male C57BL/6 mice were purchased from Shanghai Laboratory Animal Center of China Academy of Science (Shanghai, China). Mice used were between 5-8 weeks of age, and maintained in a specific pathogen-free microenvironment, and were taken care of with the guidelines outlined in the Guide for the Care and Use of Laboratory Animals. Mice were anesthetized before surgical procedure and were euthanized by suffocation with CO_2_. Animal research was approved by Animal Care and Use Committee of University of Science and Technology of China (Permit Number: USTCACUC1201003).

### Murine Mononuclear Cell (MNC) Preparation

Liver, spleen, lung, peripheral blood, mesenteric lymph node and bone marrow were collected from euthanized mice. Hepatic and pulmonary MNCs were isolated as described previously [24,25]. Briefly, livers were grinded through 200-gauge stainless steel mesh. Then the MNCs were isolated by density gradient centrifugation (750g, 30 min) with Percoll (Sigma, St Louis, MO) from the cells collected. The interphase between 40% and 70% Percoll were collected as hepatic MNCs. Lungs were minced and digested in RPMI-1640 containing 0.1% collagenase I (Sigma, St Louis, MO) and 5% fetal calf serum at 37°C for 60 min. Pulmonary MNCs were isolated by density gradient centrifugation (750g, 30 min) with Percoll. The interphase between 40% and 70% Percoll were collected as pulmonary MNCs. Spleens were grinded and passed through a 200-gauge stainless steel mesh; then the red blood cells (RBCs) were lysed. For peripheral blood MNCs, blood was collected and centrifuged for blood cells. Cells were resuspended in PBS, and the MNCs were isolated by density gradient centrifugation (750g, 30 min) with Percoll. Peripheral blood MNCs were collected from the interphase between PBS and 70% Percoll. Mesenteric lymph nodes (MLNs) were grinded with the frosted surface of microscope slides and the MNCs were collected by centrifugation. The marrow cavities of mouse femurs were lavaged with PBS and the bone marrow MNCs were collected by centrifugation; and the RBCs were lysed.

### Kupffer cell and Hepatocyte Isolation

Hepatic Kupffer cell isolation was performed in two-step collagenase perfusion method according to previous description [6]. Briefly, Liver was perfused with solution A (Ca^2+^-Mg^2+^ free HBSS added with 0.238% HEPES, 0.067% heparin and 0.019% EGTA) and solution B (Ca^2+^-Mg^2+^-glucose free HBSS added with 0.238% HEPES, 0.056% CaCl2, 0.05% collagenase IV [Roche Diagnostics, GmbH, Mannheim, Germany], 0.05% pronase E [Roche Diagnostics, GmbH, Mannheim, Germany] and 20 µg mL^-1^ deoxyribonuclease I [Worthington, NJ, U.S.A.]). Then the cells were collected by density gradient centrifugation (800g, 15 min) with Percoll after discarding the hepatocytes by low speed centrifugation (50g, 5 min). Kupffer cells were collected from the interphase between 20% Percoll and 50% Percoll. And Kupffer cells were identified as F4/80 positive for flow cytometry analysis. Hepatocytes were also isolated in the same two-step collagenase perfusion method according to previous description [26,27]. In short, livers were perfused with solution A and solution C (DMEM added with 0.075% collagenase I [Roche Diagnostics, GmbH, Mannheim, Germany]). And the hepatocytes were collected by low speed centrifugation (50g, 5 min) and isolated by density gradient centrifugation (420g, 10 min) with DMEM and 40% Percoll. The pellet was collected as hepatocytes.

### Isolation of Alveolar Mϕ and Peritoneal Mϕ

The method of the alveolar macrophages (Mϕ) and peritoneal Mϕ isolation was performed as previously described with minor modifications [28,29]. The euthanized mice were intraperitoneally injected with 4mL ice-cold PBS, and the peritoneal lavage fluid was collected; and then the lungs were lavaged with1mL ice-cold PBS for three times, the bronchoalveolar lavage fluid (BALF) was collected. The alveolar Mϕ was collected by centrifugation from the BALF; and the peritoneal Mϕ was collected by centrifugation from the peritoneal lavage fluid.

### NK Cell Purification and Cell Culture

NK cells were purified by using the NK Cell Isolation Kit II, mouse (MiltenyiBiotec, Auburn, CA 95602, USA) under the operation instruction. Ana-1cells were purchased from the Type Culture Collection of the Chinese Academy of Science (Shanghai, China). 293A cells were purchased from the ATCC. All cells were cultured in complete medium (RPMI 1640 [for Ana-1] or DMEM [for 293A], with 10% fetal bovine serum, penicillin, and streptomycin) at 37°C, in an atmosphere of 5% CO_2_. And NK cells-Ana-1cells were coculture at 1:1 ratio for 24 hours with Poly I:C (2 µg mL^-1^). Blocking antibodies (anti-CRACC kindly provided by Dr. Andrew Veillette from IRCM; anti-NKG2D from eBioscience, U.S.A.) used in this study were all used at a final concentration of 10 µg mL^-1^.

### Flow Cytometry and Antibodies

Cells in flow cytometry detection were stained with antibodies for surface marker under standard procedure. Antibodies used for flow cytometry included FITC-conjugated anti-CD3, PE-conjugated anti-NK1.1, PE-conjugated anti-2B4, Percp-Cy5.5-conjugated anti-CD19, Percp-Cy5.5-conjugated anti-F4/80 (BD PharMingen, San Diego), monoclonal anti-mouse CRACC/SLAMF7 antibody which is used combined with a secondary antibody APC-conjugated anti-rat IgG (R&D Systems, Inc.) and Alexa647-conjugated anti-Ly108, Alexa647-conjugated anti-Ly9, Alexa647-conjugated anti-CD84 were kindly provided by Dr. Andrew Veillette (IRCM, CA).

### Poly I:C and D-GalN Stimulation

High molecular weight Poly I:C (InvivoGen, San Diego, U.S.A.) and D-GalN (Sigma Chemical Co, St. Louis, MO) were prepared in clinical degree saline for injection. For upregulating the CRACC expression, Poly I:C was administrated by intraperitoneal injection at the dose of 30 µg g^-1^ body weight. For liver injury triggering, Poly I:C was given mice by intravenous injection (1.5 µg per mouse) together with D-GalN (10 mg per mouse) injection intraperitoneally.

### Nano-particle Encapsulated siRNA Treatment

To silence the expression of CRACC on Kupffer cells and Ana-1, siRNA targeting CRACC was designed and named as siCRACC (sense: CGUGCAGAGAUUUACAGUATT; anti-sense: UACUGUAAAUCUCUGCACGTT). The siCRACC was synthetized by GenePharma (Shanghai, China). To deliver the siCRACC to Kupffer cells, a cationic lipid assisted ePEG-PLA Nano-particle was used [30]. The encapsulation of siCRACC was performed by Dr. Wang Jun (USTC, China). This nano-particle encapsulated siCRACC (80 µg per mouse) was given to mice 6 hours prior to the Poly I:C/D-GalN stimulation by intravenous injection.

### RT-PCR and Quantitative PCR

Total liver RNA and cellular RNA were extracted with Trizol Reagent (Invitrogen, USA) under standard procedure, and were used for cDNA synthesis. cDNA was used in the PCR detection and quantitative PCR detection. Both detections were performed under standard procedure. The primers used to detect the CRACC expression were as follows: β-actin: sense: ATGGATGACGATATCGCT; anti-sense: ATGAGGTAGTCTGTCAGGT; CRACC: sense: AATGGCACCTGCGTAATC; anti-sense: GTGTCATAGTCTGCGTTCT.

### cDNA and pcDNA3.0-CRACC

cDNA encoding CRACC was cloned from murine lymphocytes by RT-PCR with primer modified by adding cleavage sites of BamHI and EcoRI (sense: CGGGATCCATGGCTCGTTTCTCAAC; anti-sense: CCGGAATTCTCAAAGCTTAATGACCTTGG). For the expression of CRACC in target cells, cDNA encoding CRACC was cloned into the expression plasmid pcDNA3.0. The pcDNA3.0-CRACC was transfected to 293A cells by lipofectamine 2000 (Invitrogen, USA).

### Immune-fluorescence Analysis and Laser Scanning Confocal Microscopy

Nano-particle encapsulated FAM conjunct siRNA were injected to mice intravenously and the mice were harvested 3 hours later. The liver tissues were frozen in O.C.T. Compound (Sakura Finetek U.S.A., Torrance, CA) and sectioned at -20°C. Hepatic Kupffer cells were prepared as described above. Frozen sections of liver tissues and hepatic Kupffer cells smear were prepared for the immune-fluorescence analysis (IFA) under standard procedure. Liver tissue sections and Kupffer cells smear were stained with APC-conjugated anti-F4/80 (BD PharMingen, San Diego) and examined by LSM 710 confocal laser scanning microscope (Zeiss).

### Analysis of Liver Transaminase Activities

Liver injury caused by Poly I:C and D-GalN was surveyed by measuring the serum enzyme activities of alanine aminotransferase (ALT) with ALT reagent kit (Rong Sheng, Shanghai, China) at clinical diagnostic degree.

### ELISA for Cytokine Detection

Coculture supernatant was collected and detected with corresponding ELISA Kit (DAKEWE, China) for the TNF-α and IFN-γ.

### H&E Staining

For histopathological analysis, liver sections (7 µm) were stained with Hematoxylin-Eosin and examined by microscopy according to the previous description [6].

### Statistical Analysis

The results were analyzed by Student t test or analysis of variance where appropriate. All data were shown as mean ± standard error of the mean (SEM). P value < 0.05 was considered to be statistically significant.

## Results

### 1: CRACC was abundant on hepatic NK cells

**Figure 1:**
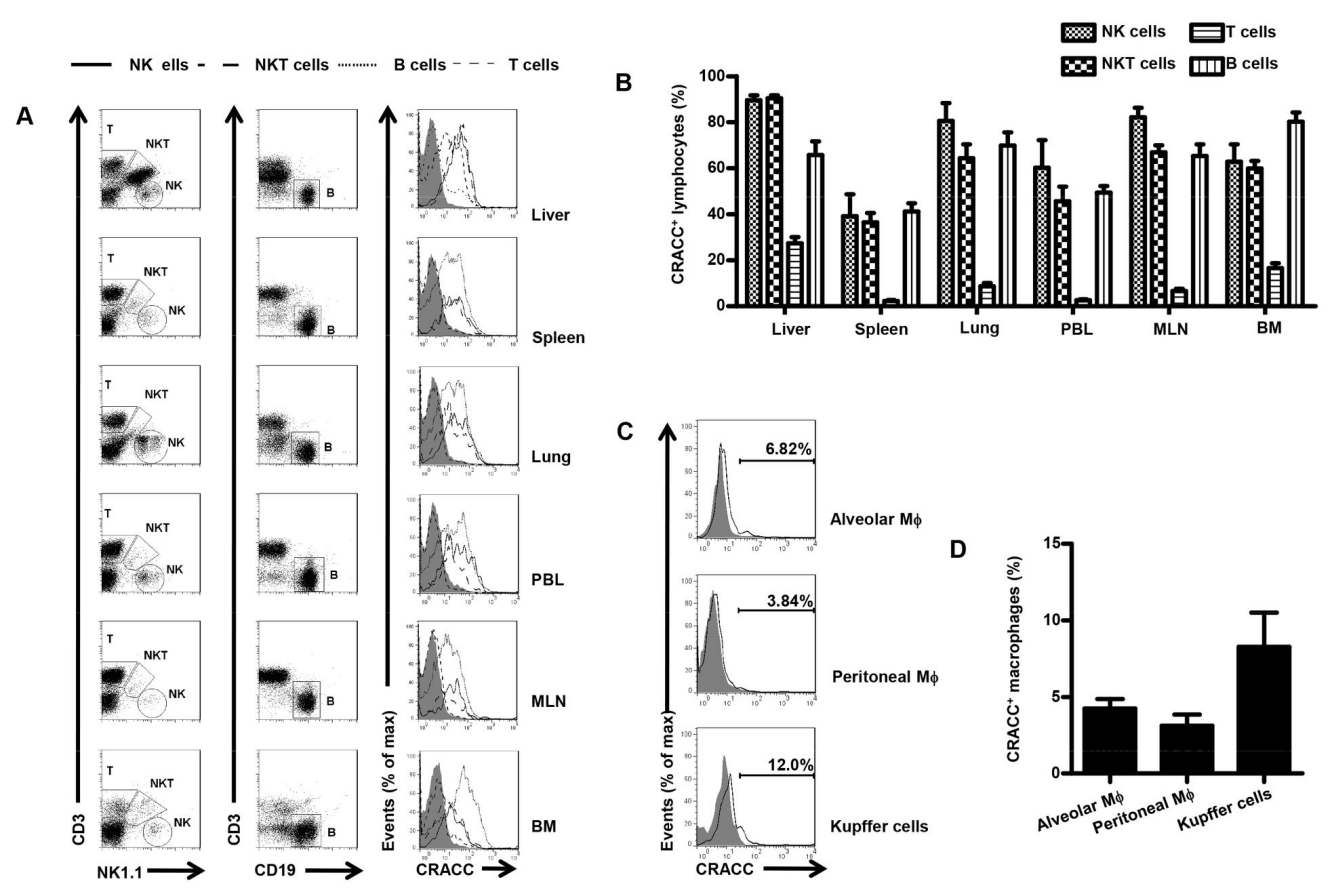
CRACC expression on lymphocytes and macrophages among various organs. **A**, Flow Cytometry of the expression of CRACC on lymphocytes (NK cells-solid line, NKT cells-long dashed line, T cells-dashed line and B cells-dotted line) from liver, spleen, lung, peripheral blood (PBL), mesenteric lymph node (MLN) and bone marrow (BM) with mCRACC mAb (open histograms) and isotype control Ab (filled histograms). NK cells were identified as NK1.1 ^+^ CD3^-^ cells; NKT cells were identified as NK1.1 ^+^ CD3^+^ cells; T cells were identified as NK1.1^-^CD3^+^ cells; B cells were identified as CD3^-^CD19^+^ cells. **B**, Percent of CRACC^+^ cells among lymphocytes from liver, spleen, lung, PBL, MLN and BM. **C**, Flow cytometry of CRACC expression on macrophages from bronchoalveolar lavage fluid (BALF; alveolar Mϕ), peritoneal cavity (peritoneal Mϕ), and liver (Kupffer cells). Macrophages were identified as F4/80^+^ cells. **D**, Percent of CRACC^+^ cells among alveolar Mϕ, peritoneal Mϕ and Kupffer cells. Data are from 6-8 mice per group.

At the beginning of this study, we screened the murine CRACC expression patterns among NK cells, NKT cells, T cells and B cells from the liver, spleen, lung, peripheral blood (PBL), mesenteric lymph node (MLN) and bone marrow (BM). We found higher levels of CRACC expression on NK cells and NKT cells, compared with that on T cells and B cells. The CRACC expression was different on the same type of lymphocytes among different organs. CRACC levels on hepatic lymphocytes were much higher compared with those on splenic and peripheral lymphocytes (Figure 1A, B). These data show that CRACC is dominantly expressed on innate immunity associated lymphocytes in the liver, which is also known as an innate immune associated organ. Since Kupffer cells, which are liver resident macrophages (Mϕ), are also critical innate immune cells, we screened the CRACC expression patterns on Kupffer cells as well as on other Mϕ from BALF and peritoneal cavity. Compared to NK and NKT cells, macrophages had much lower levels of CRACC expression (Figure 1). Expression of CRACC on Kupffer cells was higher than that on alveolar Mϕ and peritoneal Mϕ (Figure 1C, D).

### 2: Up-regulation of CRACC expression on NK cells and Kupffer cells by Poly I:C injection

**Figure 2:**
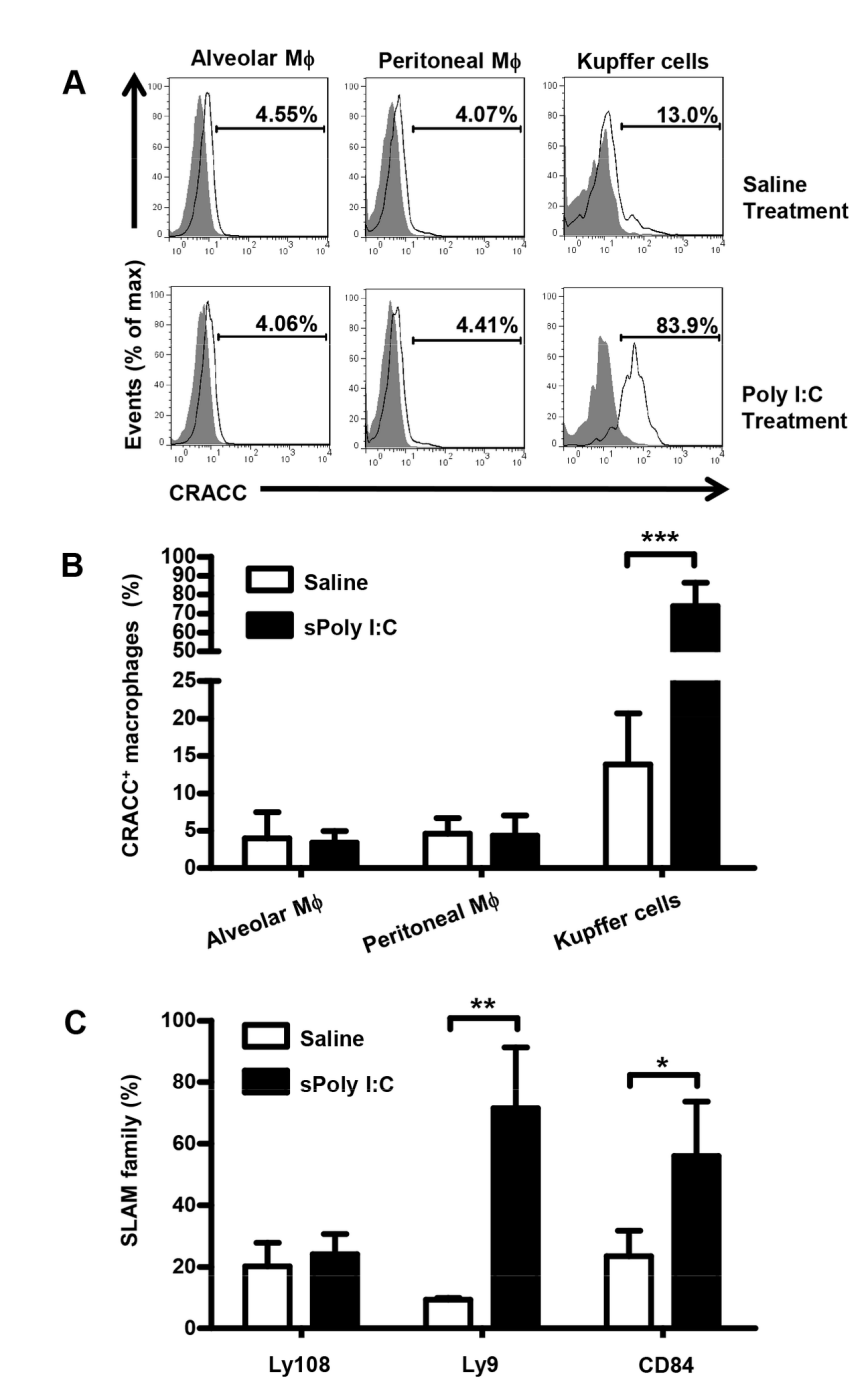
Poly I:C injection elevates the expression of CRACC on Kupffer cells. CRACC expression on alveolar Mϕ, peritoneal Mϕ and Kupffer cells from mice treated with Poly I:C for 18h was analyzed by flow cytometry. Alveolar Mϕ, peritoneal Mϕ and Kupffer cells were identified as F4/80^+^ cells. **A**, Flow Cytometry of the CRACC expression on alveolar Mϕ, peritoneal Mϕ and Kupffer cells. **B**, Percent of CRACC^+^ cells among alveolar Mϕ, peritoneal Mϕ and Kupffer cells. **C**, Percent of SLAM family (Ly108, Ly9 and CD84) positive cells among Kupffer cells from mice treated with Poly I:C for 18h. Data are from 6-9 mice per group. *P < 0.05; **P<0.01; ***P < 0.001.

CRACC expression on Mϕ has not been determined so far. This might be due to the low degree of CRACC expression on Mϕ under normal conditions (Figure 1C, D). However we found that the expression of CRACC on Kupffer cells was elevated dramatically by Poly I:C injection, while no significant change was observed on alveolar Mϕ and peritoneal Mϕ (Figure 2A, B). Then we tried to figure out whether the elevated expression by Poly I:C treatment was restricted to CRACC among the SLAM family. We detected the expression of Ly108, Ly9, and CD84 on Kupffer cells and found the Ly9, CD84 expressions on Kupffer cells were also upregulated by Poly I:C injection besides CRACC (Figure 2C).

**Figure 3:**
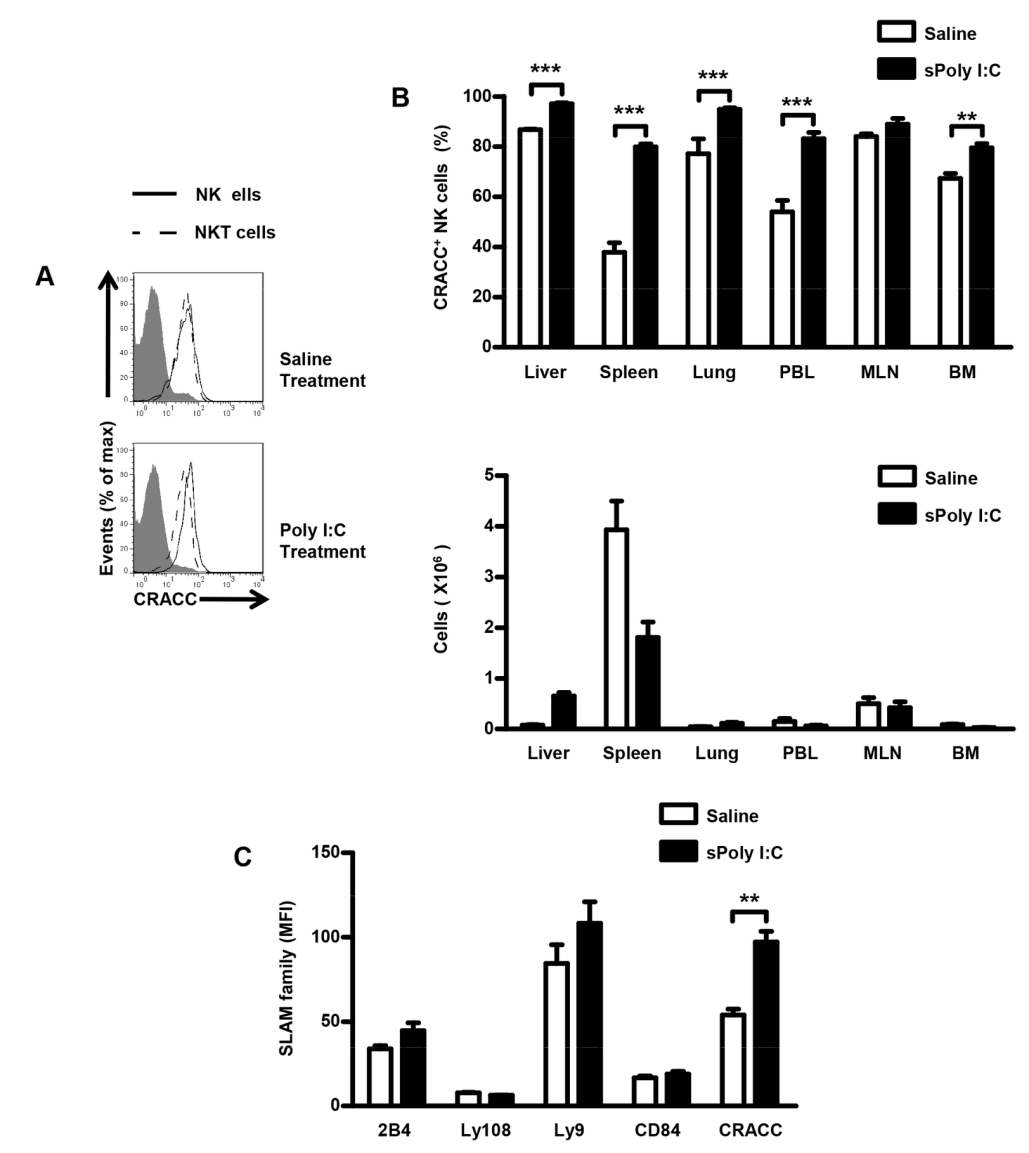
Poly I:C treatment up regulates the expression of CRACC on NK cells. CRACC expression on NK cells (solid line) and NKT cells (long dashed line) from mice treated with Poly I:C for 18h was analyzed by flow cytometry. NK cells were identified as NK1.1 ^+^ CD3^-^ cells and NKT cells were identified as NK1.1 ^+^ CD3^+^ cells. **A**, Flow Cytometry of the CRACC expression on hepatic NK cells and hepatic NKT cells. **B**, *Upper* *panel*: percent of CRACC^+^ cells among NK cells from liver, spleen, lung, peripheral blood (PBL), mesenteric lymph nodes (MLN), and bone marrow (BM); *Lower* *panel*: cells counts of NK cells from indicated organs. **C**, MFI analyses of SLAM family (2B4, Ly108, Ly9, CD84, CRACC) expression on hepatic NK cells from mice treated with Poly I:C for 18h by flow cytometry. Data are from 5-8 mice per group. **P<0.01; ***P < 0.001.

Since it was reported that Poly I:C treatment upregulated the expression of CRACC on splenic NK cells [14], we tried to find out whether the CRACC expression on hepatic NK cells is also upregulated by Poly I:C injection. We analyzed the effect of Poly I:C injection to the expression of CRACC on NK cells among different organs, and found remarkably upregulated expression on hepatic NK cells (Figure 3A, B). In contrast, expression of CRACC on liver NKT cells was unchanged after Poly I:C treatment (Figure 3A). Besides CRACC, we also examined the effect of Poly I:C injection to the expression of other SLAM family members (2B4, Ly108, Ly9 and CD84) on hepatic NK cells. The data showed that only the expression of CRACC on NK cells was upregulated significantly while the expression of other SLAM family members remained unchanged after Poly I:C treatment (Figure 3C).

**Figure 4:**
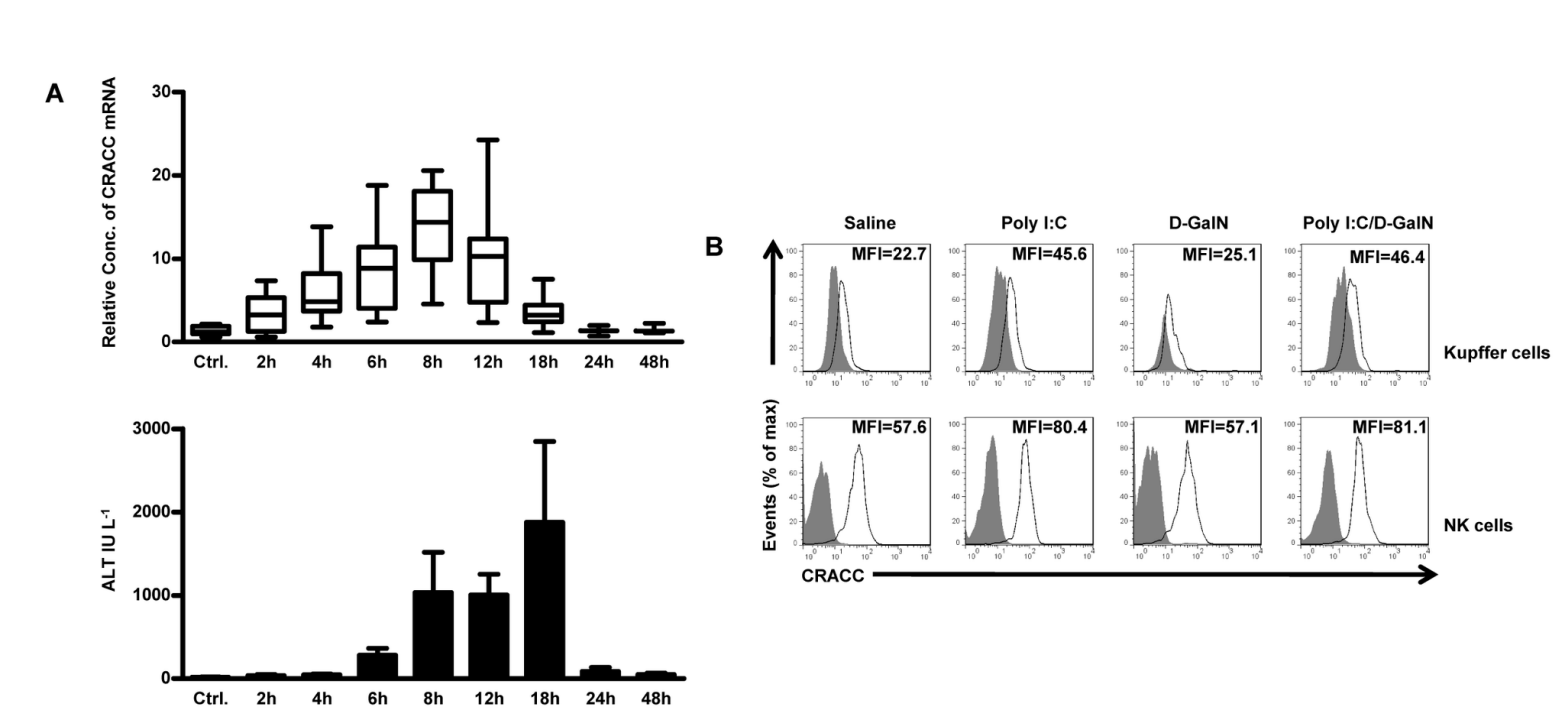
Poly I:C/D-GalN induced liver injury correlates to the increased CRACC mRNA expression of liver. **A**, Mice were treated with Poly I:C (1.5 µg per mouse, i.v.) together with D-GalN (10 mg per mouse, i.p.); and then euthanized at 2h, 4h, 6h, 8h, 12h, 18h, 24h, 48h time points post Poly I:C/D-GalN treatment. The expression of CRACC mRNA was assayed by quantitative PCR (upper panel); and the serum ALT was tested (lower panel). **B**, Mice were treated with saline alone, Poly I:C alone, D-GalN alone or Poly I:C together with D-GalN for 12h; and then the CRACC expression on Kupffer cells and hepatic NK cells was analyzed by flow cytometry. Kupffer cells were identified as F4/80^+^ cells and NK cells were identified as NK1.1 ^+^ CD3^-^ cells. Data are from 6-9 mice per group.

Our previous study has established a classical NK cell-mediated acute liver injury model induced by Poly I:C and D-GalN. It was proved that the interaction of NKG2D and its ligand Rae-1 between NK cells and Kupffer cells was responsible to this injury. Because the blockade of NKG2D did not prevent the injury completely, we hypothesized that there are some other co-stimulating molecules involved in inducing liver injury. The elevated expression of CRACC on Kupffer cells and NK cells by Poly I:C implied a potential promoting role of CRACC in this pathogenic model. Then we examined the expression of CRACC in liver tissues from mice treated with Poly I:C and D-GalN dynamically. We found that hepatic expression of CRACC was upregulated dynamically, and the serum ALT elevated subsequently; and the peak levels of CRACC expression appeared prior to the serum ALT peak value (Figure 4A).

The above data revealed that Poly I:C injection upregulated CRACC expression in both hepatic NK cells and Kupffer cells. Next we examined CRACC expression on hepatic NK cells and Kupffer cells in the Poly I:C/D-GalN model. The data confirmed that the expression of CRACC was upregulated on Kupffer cells and NK cells by Poly I:C/D-GalN treatment, which is similar to the results by Poly I:C alone; while the D-GalN alone had no such effect (Figure 4B).

Expression of SLAM family is usually restricted to hemopoietic cells. Because the fetal liver is a hemopoietic organ, we wondered whether hepatocytes also expressed CRACC. However, our data revealed that the expression of CRACC was undetectable at neither transcriptional levels nor membrane protein levels on hepatocytes, and Poly I:C stimulation did not evoke the expression (Figure S1A, B). Therefore, these findings ruled out the possibility that hepatocytes expressed CRACC as ligand.

Collectively, our data revealed that Poly I:C injection upregulated the expression of CRACC on both Kupffer cells and NK cells; and the increased CRACC expression in the liver was correlated with Poly I:C/D-GalN-induced liver injury. This suggests that the CRACC may play a role in promoting the liver injury model induced by Poly I:C/D-GalN.

### 3: Kupffer cell-specific silence of CRACC alleviates NK cell-mediated liver injury

**Figure 5:**
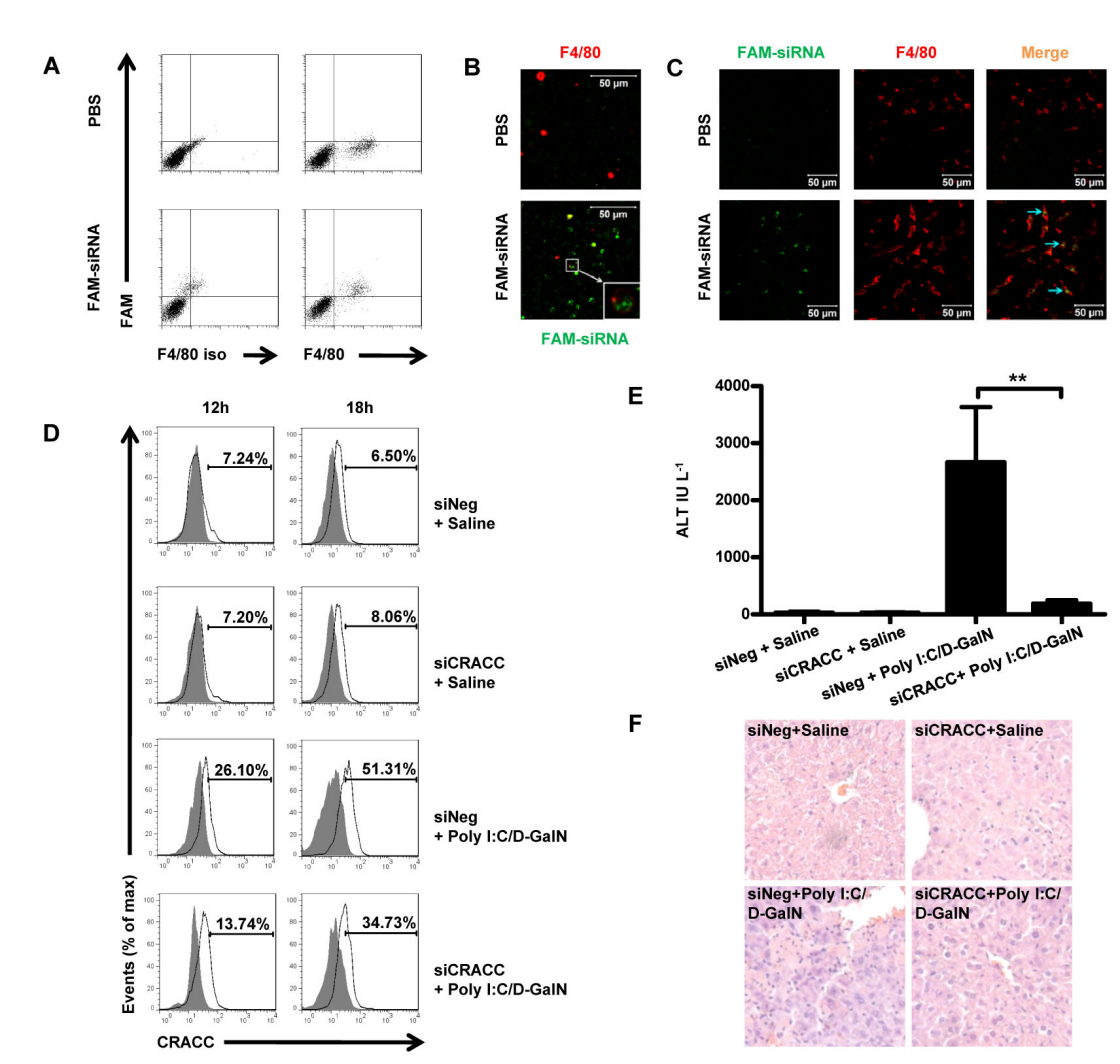
Silencing CRACC expression on Kupffer cells alleviates the liver injury induced by Poly I:C/D-GalN. **A**-**C**, Mice were treated with nanoparticle encapsulated FAM conjunct siRNA by intravenous injection; and 3h later FAM^+^ cells among Kupffer cells were analyzed by flow cytometry (**A**); and the nanoparticle encapsulated FAM conjunct siRNA (green) endocytosed by Kupffer cells (red, stained with APC-F4/80 mAb) were tracked by con-focal microscopy both in isolated Kupffer cells (**B**, Bar=50µm, 63× objective) and in site of frozen tissue sections(**C**, Bar=50µm, 40× objective). Kupffer cells were identified as F4/80^+^ cells. **D**, Mice were pre-treated with nanoparticle encapsulated siNeg or siCRACC for 6h, and then treated with Poly I:C/D-GalN in each group. The CRACC expression on Kupffer cells was analyzed by flow cytometry at 12h and 18h time points post the Poly I:C/D-GalN treatment. The Data are from 6-8 mice per group. **E**-**F**, The mice were pre-treated with nanoparticle encapsulated siNeg or siCRACC for 6h, and then treated with Poly I:C/D-GalN in each group. The serum ALT was tested (**E**) and the H&E-Staining analyses were performed at 18h time point post Poly I:C/D-GalN (**F**). The Data are from 6-8 mice per group. **P < 0.01.

To determine the role of CRACC in Poly I:C/D-GalN-induced liver injury, the expression of CRACC on Kupffer cells was specifically suppressed by RNAi. To deliver the siRNA to Kupffer cells specifically, a novel nanoparticle encapsulated siRNA administration method was employed. We designed a pair of siRNA targeting at CRACC and named it as siCRACC (Figure S3). The siRNA was encapsulated by cationic lipid assisted mPEG-PLA nanoparticle, and given to mice by common intravenous injection. It proved the capacity of this kind of nanoparticle delivering the siRNA to Kupffer cells specifically by both flow cytometry detection and Laser Scanning Confocal Microscope (LSCM) imaging. The flow cytometry data revealed that almost all the Kupffer cells phagocytosed this nanoparticle encapsulating FAM conjunct siRNA (Figure 5A). LSCM imaging analyses further confirmed this nanoparticle was phagocytosed by Kupffer cells specifically (Figure 5B, C). Administration of this particle did not cause obvious liver injury (Figure S2).

Then the mice were pre-transfected with nanoparticle encapsulated siCRACC 6h prior to the Poly I:C/D-GalN treatment. We found the CRACC expression on Kupffer cells was downregulated by siCRACC (Figure 5D) 12h-18h post the Poly I:C/D-GalN treatment, and the liver injury was alleviated significantly (Figure 5E, F). These findings indicate that blockade of CRACC expression on Kupffer cells alleviates Poly I:C/D-GalN-induced liver injury, suggesting that upregulation of CRACC on Kupffer cells contributes to such injury.

### 4: CRACC-CRACC interaction between NK cells and Kupffer cells is critical in NK cell activation

Our previous studies demonstrated that Poly I:C/D-GalN-induced liver injury was mediated by TNF-α and IFN-γ, which were produced during the NK-Kupffer cell interaction, herein we focused on the effect of CRACC to the TNF-α and IFN-γ production. To further confirm the mechanism of CRACC enhancing liver injury, we established a coculture system of primary NK cells and a murine macrophage cell line-Ana-1 in vitro. The system was used to imitate the Kupffer-NK cell interaction.

**Figure 6:**
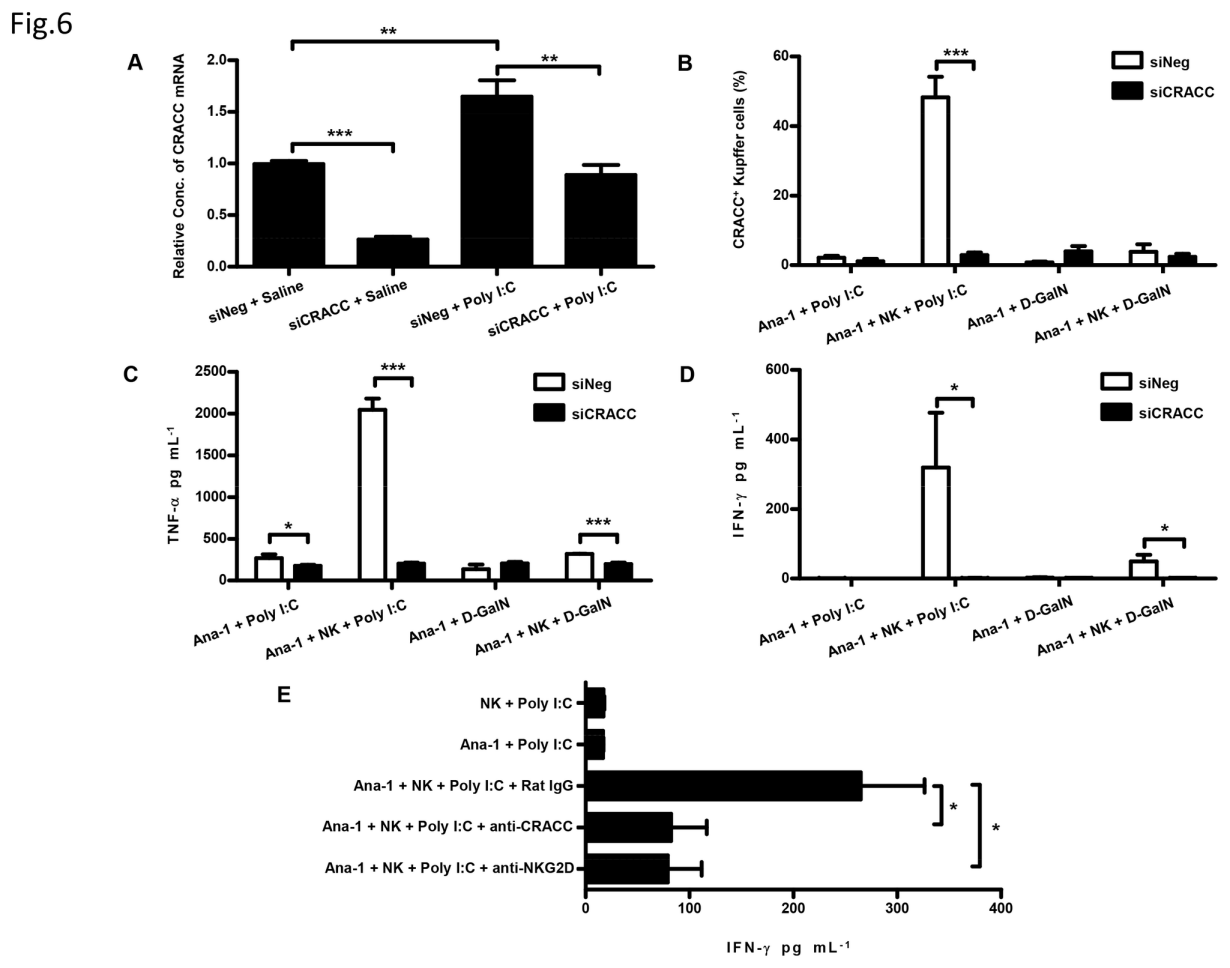
CRACC-CRACC interaction between macrophages and NK cells enhances the cytokine secretion in vitro. **A**, Ana-1 cells pre-transfected with liposome encapsulated siNeg or siCRACC was subsequently stimulated with Poly I:C. The expression of CRACC mRNA was assayed by quantitative PCR at 3h time point post the Poly I:C stimulation. **B**, **C** & **D**, Ana-1 cells pre-transfected with nanoparticle encapsulated siNeg or siCRACC was cultured in absence or presence of NK cells, which were freshly isolated from mice pre-treated by Poly I:C. Then Ana-1 cells cultured with or without NK cells were stimulated with Poly I:C; and D-GalN was made as control. CRACC expression on Ana-1 cells was analyzed by flow cytometry (**B**) and the cytokine (TNF-α and IFN-γ) in the culture supernatant was assayed by ELISA (**C**, **D**) at 24h time point post the Poly I:C or D-GalN stimulation; Ana-1 cells were identified as F4/80^+^ cells. **E**, Ana-1 cells were cocultured with freshly isolated NK cells from mice pre-treated by Poly I:C; and Ana-1 cells alone or NK cells alone were made as control. The Ana-1-NK coculture was stimulated by Poly I:C in the presence of isotype control Ab (Rat IgG), CRACC mAb (anti-CRACC), or NKG2D mAb (anti-NKG2D), respectively. The IFN-γ in culture supernatant was assayed by ELISA at 24h time point post the Poly I:C stimulation. *P < 0.05; **P<0.01; ***P < 0.001.

First, we proved the CRACC expression pattern on Ana-1 was comparable with Kupffer cells. It was found that the Ana-1 expressed very low levels of CRACC under normal conditions, but Poly I:C stimulation markedly upregulated the expression. Moreover, the expression of CRACC on Ana-1 is downregulated by siCRACC treatment (Figure 6A).

Next, we stimulated the NK-Ana-1coculture system with Poly I:C for 24h with or without silencing the CRACC expression on Ana-1. As illustrated in Figure 6B-D, silence of CRACC expression on Ana-1 cells significantly inhibited TNF-α and IFN-γ secretion in the co-cultures. To confirm whether CRACC-CRACC interaction between Ana-1 and NK cells increased the cytokine production, we also used a CRACC monoclonal antibody to block the CRACC-CRACC interaction in this coculture system. Our data in Figure 6E revealed that IFN-γ production was decreased in the group treated with the CRACC antibody (Figure 6E). In agreement with previous findings, treatment with an NKG2D antibody also inhibited IFN-γ production.

These data implies that CRACC-CRACC interaction between Kupffer cells and NK cells might enhance the Poly I:C/D-GalN induced liver injury by increasing cytokine production.

## Discussion

**Figure 7:**
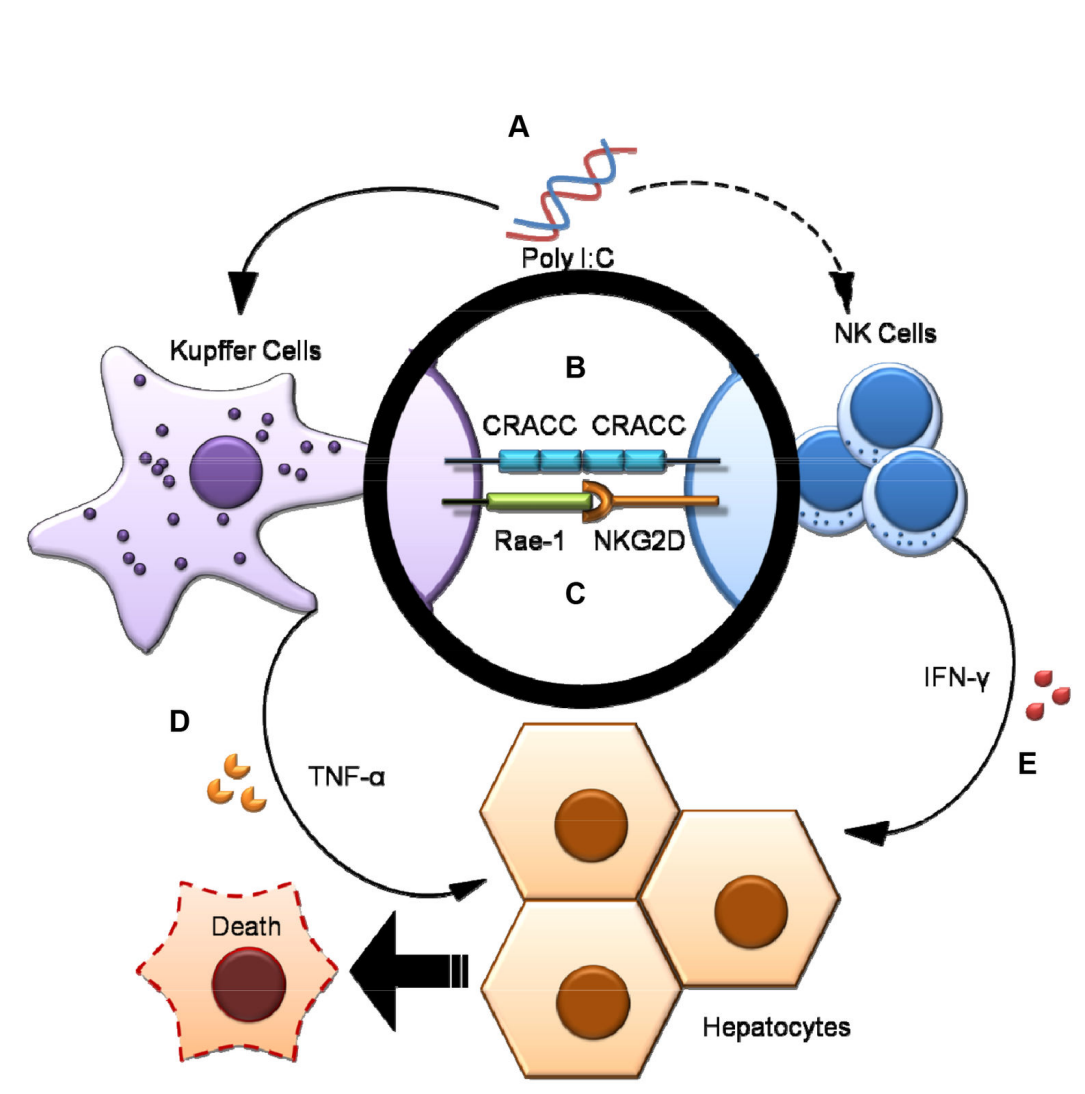
CRACC-CRACC interaction between NK cells and resident Kupffer cells. Poly I:C stimulation (**A**) elevated the CARCC expression on NK cells and resident Kupffer cells (**B**). CRACC-CRACC interaction (**B**) together with the NKG2D-Rae-1 interaction (**C**) between NK cells and resident Kupffer cells promote IFN-γ and TNF-α production (**D**, **E**), which contributes to the Poly I:C/D-GalN induced liver injury.

This study has demonstrated that CRACC-CRACC interaction between hepatic NK cells and Kupffer cells plays an important role in promoting the Poly I:C/D-GalN induced liver injury. Poly I:C treatment upregulated the expression of CRACC on Kupffer cells as well as on NK cells; and the CRACC interaction between NK cells and Kupffer cells upregulated the secretion of IFN-γ and TNF-α, thereby contributing to liver injury (Figure 7). By using this model, we have demonstrated that the SLAM family receptors participate in the immunomodulation among hepatic innate immune cells in hepatitis.

The broad expression of CRACC on various types of immune cells has been reported previously, however we firstly screen the expression pattern on multiple lymphocytes from different organs. In our study, we found different CRACC expression pattern among NK cells from different organs. CRACC tends to be expressed at higher levels on liver NK cells, compared with the peripheral and splenic NK cells. This might be one reflection of the different immunophenotypical features between the liver-specific NK cells and the peripheral and spleen NK cells. It is believed that the liver-specific NK cells display higher cytotoxicity against tumor cells by the immunophenotypical, morphological, and functional characteristics [31]. Since CRACC is proved a critical activating receptor to the NK cells cytotoxicity previously [14], the higher levels of CRACC expressed on liver NK cells may contribute to the higher cytotoxicity activities of liver-specific NK cells compared with peripheral NK cells.

The expression of CRACC on macrophages has not been previously determined. This might due to two possible reasons: the low levels of CRACC expression on macrophages under normal conditions and the phenotypical difference of macrophages from different tissues. CRACC and the other SLAM family members are usually expressed on hematopoietic cells at high levels to exert immunomodulatory function [13,15,16]. However, our data revealed very low levels of CRACC expression on macrophages with slightly higher levels on Kupffer cells compared with that on macrophages from BALF and peritoneal cavity. Such low levels may not act as a functional receptor. However, after Poly I:C treatment, expression of CRACC on macrophages was markedly upregulated and may act as an activating receptor to induce NK cell activation.

There are other members of SLAMs expressed on macrophages as well as on NK cells. And current studies reported some of them played positive regulatory roles in macrophages and NK cells [32–34]. Besides CRACC, our data revealed that the expressions of Ly9 and CD84 on Kupffer cells were also elevated by Poly I:C stimulation. However, only the expression of CRACC was upregulated on both NK cells and Kupffer cells by Poly I:C. Otherwise, it is known that the activating signaling by CRACC in NK cells depends on the expression of EAT-2 (Ewing’s sarcoma-activated transcript 2), which is abundant in NK cells, while the activating signaling by Ly9 and CD84 depends on SAP, which is absence in macrophage so far as we know [16]. Thus, the altered expression of these receptors remains unclear. More efforts are in need to reveal the functions of these receptors on NK cells and Kupffer cells.

In this study, we demonstrated that Poly I:C treatment markedly upregulated the expression of CRACC on Kupffer cells. Since Poly I:C is known as mimic of double strands RNA virus, it implied that the CRACC expression on macrophages can be induced by certain PAMPs stimulation under infection conditions, which may promotes the anti-virus immunity or leads to immune injury. However, CRACC expressed on other macrophages did not respond to Poly I:C stimulation in our experiments. This might be due to the special local microenvironment of livers for Kupffer cells, such as cytokines, hepatocytes, and other liver nonparaenchymal cells. The mechanism remains to be further investigated.

The functions of CRACC to NK cells are well known as an activating receptor of cytotoxicity. Our experiments demonstrated that CRACC activation enhanced the IFN-γ production of NK cells in Poly I:C/D-GalN induced liver injury. This implies CRACC on NK cells not only acts a cytotoxicity activating receptor but also acts a cytokine production promoting receptor. The in vitro experiments in the current study proved the silencing of CRACC expression on macrophages reduced the TNF-α production. This suggests that the CRACC on macrophages acts an activating receptor to enhance the TNF-α production. Collectively, our findings suggest that CRACC-CRACC interaction between Kupffer and hepatic NK cells promotes cytokine production, which contributes to Poly I:C/D-GalN induced liver injury. These data are supplemental to the cellular cross-talk between Kupffer and hepatic NK cells mediated by NKG2D-Rae-1 in our previous study. In the current study, we have also demonstrated for the first time that SLAM family member CRACC participates in the liver immunomodulation via NK-Kupffer interaction and promotes liver injury. Further studies to characterize the molecular mechanism of CRACC-CRACC interaction between NK and Kupffer cells will help us understand better the pathogenesis of immune-mediated liver injury.

## Supporting Information

Figure S1 **CRACC is undetectable on hepatocytes.** **A**, CRACC mRNA expression of murine hepatocytes was assayed by RT-PCR. **B**, Mice were treated with Poly I:C, and CRACC expression on hepatocytes was analyzed by flow cytometry at 18h time point post the Poly I:C treatment.
(TIF)

Figure S2 **Nanoparticle encapsulated FAM conjunct siRNA induces no liver injury.** Mice were treated with nanoparticle encapsulated FAM conjunct siRNA by intravenous injection; and the serum ALT was tested 3h later.
(TIF)

Figure S3 **The CRACC expression is silenced by siCRACC.** 293A cells were transfected with pcDNA3.0-CRACC together with siNeg or siCRACC. The expression of CRACC on 293A cells was assayed by quantitative PCR at 12h, 24h, 36h and 48h time points.
(TIF)

## References

[1] Tian Z, Chen Y, Gao B (2013) Natural killer cells in liver disease. Hepatology 57: 1654-1662. doi:10.1002/hep.26115. PubMed: 23111952. 23111952 10.1002/hep.26115 PMC3573257

[2] Miyazawa Y, Tsutsui H, Mizuhara H, Fujiwara H, Kaneda K (1998) Involvement of intrasinusoidal hemostasis in the development of concanavalin A-induced hepatic injury in mice. Hepatology 27: 497-506. doi:10.1002/hep.510270225. PubMed: 9462649. 9462649 10.1002/hep.510270225

[3] Biburger M, Tiegs G (2005) Alpha-galactosylceramide-induced liver injury in mice is mediated by TNF-alpha but independent of Kupffer cells. J Immunol 175: 1540-1550. PubMed: 16034092. 16034092 10.4049/jimmunol.175.3.1540

[4] Galanos C, Freudenberg MA, Reutter W (1979) Galactosamine-induced sensitization to the lethal effects of endotoxin. Proc Natl Acad Sci U S A 76: 5939-5943. doi:10.1073/pnas.76.11.5939. PubMed: 293694. 293694 10.1073/pnas.76.11.5939 PMC411768

[5] Dong Z, Wei H, Sun R, Hu Z, Gao B et al. (2004) Involvement of natural killer cells in PolyI:C-induced liver injury. J Hepatol 41: 966-973. doi:10.1016/j.jhep.2004.08.021. PubMed: 15582130. 15582130 10.1016/j.jhep.2004.08.021

[6] Hou X, Zhou R, Wei H, Sun R, Tian Z (2009) NKG2D-retinoic acid early inducible-1 recognition between natural killer cells and Kupffer cells in a novel murine natural killer cell-dependent fulminant hepatitis. Hepatology 49: 940-949. doi:10.1002/hep.22725. PubMed: 19177594. 19177594 10.1002/hep.22725

[7] Zhou Z, Zhang C, Zhang J, Tian Z (2012) Macrophages help NK cells to attack tumor cells by stimulatory NKG2D ligand but protect themselves from NK killing by inhibitory ligand Qa-1. PLOS ONE 7: e36928. doi:10.1371/journal.pone.0036928. PubMed: 22629344. 22629344 10.1371/journal.pone.0036928 PMC3356357

[8] Cannons JL, Qi H, Lu KT, Dutta M, Gomez-Rodriguez J et al. (2010) Optimal germinal center responses require a multistage T cell:B cell adhesion process involving integrins, SLAM-associated protein, and CD84. Immunity 32: 253-265. doi:10.1016/j.immuni.2010.01.010. PubMed: 20153220. 20153220 10.1016/j.immuni.2010.01.010 PMC2830297

[9] Qi H, Cannons JL, Klauschen F, Schwartzberg PL, Germain RN (2008) SAP-controlled T-B cell interactions underlie germinal centre formation. Nature 455: 764-769. doi:10.1038/nature07345. PubMed: 18843362. 18843362 10.1038/nature07345 PMC2652134

[10] Réthi B, Gogolák P, Szatmari I, Veres A, Erdôs E et al. (2006) SLAM/SLAM interactions inhibit CD40-induced production of inflammatory cytokines in monocyte-derived dendritic cells. Blood 107: 2821-2829. doi:10.1182/blood-2005-06-2265. PubMed: 16317102. 16317102 10.1182/blood-2005-06-2265 PMC1895370

[11] Waggoner SN, Kumar V (2012) Evolving role of 2B4/CD244 in T and NK cell responses during virus infection. Front Immunol 3: 377 PubMed: 23248626. 23248626 10.3389/fimmu.2012.00377 PMC3518765

[12] Wu Y, Kuang DM, Pan WD, Wan YL, Lao XM et al. (2013) Monocyte/macrophage-elicited natural killer cell dysfunction in hepatocellular carcinoma is mediated by CD48/2B4 interactions. Hepatology 57: 1107-1116. doi:10.1002/hep.26192. PubMed: 23225218. 23225218 10.1002/hep.26192

[13] Cannons JL, Tangye SG, Schwartzberg PL (2011) SLAM family receptors and SAP adaptors in immunity. Annu Rev Immunol 29: 665-705. doi:10.1146/annurev-immunol-030409-101302. PubMed: 21219180. 21219180 10.1146/annurev-immunol-030409-101302

[14] Cruz-Munoz ME, Dong Z, Shi X, Zhang S, Veillette A (2009) Influence of CRACC, a SLAM family receptor coupled to the adaptor EAT-2, on natural killer cell function. Nat Immunol 10: 297-305. doi:10.1038/ni.1693. PubMed: 19151721. 19151721 10.1038/ni.1693

[15] Veillette A (2006) Immune regulation by SLAM family receptors and SAP-related adaptors. Nat Rev Immunol 6: 56-66. doi:10.1038/nri1761. PubMed: 16493427. 16493427 10.1038/nri1761

[16] Veillette A (2010) SLAM-family receptors: immune regulators with or without SAP-family adaptors. Cold Spring Harb Perspect Biol 2: a002469. doi:10.1101/cshperspect.a002469. PubMed: 20300214. 20300214 10.1101/cshperspect.a002469 PMC2829957

[17] Tassi I, Colonna M (2005) The cytotoxicity receptor CRACC (CS-1) recruits EAT-2 and activates the PI3K and phospholipase Cgamma signaling pathways in human NK cells. J Immunol 175: 7996-8002. PubMed: 16339536. 16339536 10.4049/jimmunol.175.12.7996

[18] Kim JR, Mathew SO, Patel RK, Pertusi RM, Mathew PA (2010) Altered expression of signalling lymphocyte activation molecule (SLAM) family receptors CS1 (CD319) and 2B4 (CD244) in patients with systemic lupus erythematosus. Clin Exp Immunol 160: 348-358. doi:10.1111/j.1365-2249.2010.04116.x. PubMed: 20345977. 20345977 10.1111/j.1365-2249.2010.04116.x PMC2883105

[19] Fasth AE, Björkström NK, Anthoni M, Malmberg KJ, Malmström V (2010) Activating NK-cell receptors co-stimulate CD4(+)CD28(-) T cells in patients with rheumatoid arthritis. Eur J Immunol 40: 378-387. doi:10.1002/eji.200939399. PubMed: 19904767. 19904767 10.1002/eji.200939399

[20] Hsi ED, Steinle R, Balasa B, Szmania S, Draksharapu A et al. (2008) CS1, a potential new therapeutic antibody target for the treatment of multiple myeloma. Clin Cancer Res 14: 2775-2784. doi:10.1158/1078-0432.CCR-07-4246. PubMed: 18451245. 18451245 10.1158/1078-0432.CCR-07-4246 PMC4433038

[21] Krämer B, Kebschull M, Nowak M, Demmer RT, Haupt M et al. (2013) Role of the NK cell-activating receptor CRACC in periodontitis. Infect Immun 81: 690-696. doi:10.1128/IAI.00895-12. PubMed: 23250953. 23250953 10.1128/IAI.00895-12 PMC3584889

[22] Schroeder A, Levins CG, Cortez C, Langer R, Anderson DG (2010) Lipid-based nanotherapeutics for siRNA delivery. J Intern Med 267: 9-21. doi:10.1111/j.1365-2796.2009.02189.x. PubMed: 20059641. 20059641 10.1111/j.1365-2796.2009.02189.x PMC5308083

[23] Shi B, Keough E, Matter A, Leander K, Young S et al. (2011) Biodistribution of small interfering RNA at the organ and cellular levels after lipid nanoparticle-mediated delivery. J Histochem Cytochem 59: 727-740. doi:10.1369/0022155411410885. PubMed: 21804077. 21804077 10.1369/0022155411410885 PMC3261601

[24] Wang J, Sun R, Wei H, Dong Z, Gao B et al. (2006) Poly I:C prevents T cell-mediated hepatitis via an NK-dependent mechanism. J Hepatol 44: 446-454. doi:10.1002/hep.21272. PubMed: 16310275. 16310275 10.1016/j.jhep.2005.08.015

[25] Ishikawa E, Nakazawa M, Yoshinari M, Minami M (2005) Role of tumor necrosis factor-related apoptosis-inducing ligand in immune response to influenza virus infection in mice. J Virol 79: 7658-7663. doi:10.1128/JVI.79.12.7658-7663.2005. PubMed: 15919918. 15919918 10.1128/JVI.79.12.7658-7663.2005 PMC1143624

[26] Seglen PO (1976) Preparation of isolated rat liver cells. Methods Cell Biol 13: 29-83. doi:10.1016/S0091-679X(08)61797-5. PubMed: 177845. 177845 10.1016/s0091-679x(08)61797-5

[27] Chen M, Tabaczewski P, Truscott SM, Van Kaer L, Stroynowski I (2005) Hepatocytes express abundant surface class I MHC and efficiently use transporter associated with antigen processing, tapasin, and low molecular weight polypeptide proteasome subunit components of antigen processing and presentation pathway. J Immunol 175: 1047-1055. PubMed: 16002705. 16002705 10.4049/jimmunol.175.2.1047

[28] Li F, Zhu H, Sun R, Wei H, Tian Z (2012) Natural killer cells are involved in acute lung immune injury caused by respiratory syncytial virus infection. J Virol 86: 2251-2258. doi:10.1128/JVI.06209-11. PubMed: 22171263. 22171263 10.1128/JVI.06209-11 PMC3302418

[29] Choi HG, Lee DS, Li B, Choi YH, Lee SH et al. (2012) Santamarin, a sesquiterpene lactone isolated from Saussurea lappa, represses LPS-induced inflammatory responses via expression of heme oxygenase-1 in murine macrophage cells. Int Immunopharmacol 13: 271-279. doi:10.1016/j.intimp.2012.04.016. PubMed: 22564506. 22564506 10.1016/j.intimp.2012.04.016

[30] Yang XZ, Dou S, Sun TM, Mao CQ, Wang HX et al. (2011) Systemic delivery of siRNA with cationic lipid assisted PEG-PLA nanoparticles for cancer therapy. J Control Release 156: 203-211. doi:10.1016/j.jconrel.2011.07.035. PubMed: 21839126. 21839126 10.1016/j.jconrel.2011.07.035

[31] Gao B, Radaeva S, Park O (2009) Liver natural killer and natural killer T cells: immunobiology and emerging roles in liver diseases. J Leukoc Biol 86: 513-528. PubMed: 19542050. 19542050 10.1189/jlb.0309135 PMC2735282

[32] Sintes J, Romero X, de Salort J, Terhorst C, Engel P (2010) Mouse CD84 is a pan-leukocyte cell-surface molecule that modulates LPS-induced cytokine secretion by macrophages. J Leukoc Biol 88: 687-697. doi:10.1189/jlb.1109756. PubMed: 20628063. 20628063 10.1189/jlb.1109756 PMC6608011

[33] Wang N, Satoskar A, Faubion W, Howie D, Okamoto S et al. (2004) The cell surface receptor SLAM controls T cell and macrophage functions. J Exp Med 199: 1255-1264. doi:10.1084/jem.20031835. PubMed: 15123745. 15123745 10.1084/jem.20031835 PMC2211908

[34] Tangye SG, Phillips JH, Lanier LL, Nichols KE (2000) Functional requirement for SAP in 2B4-mediated activation of human natural killer cells as revealed by the X-linked lymphoproliferative syndrome. J Immunol 165: 2932-2936. PubMed: 10975798. 10975798 10.4049/jimmunol.165.6.2932

