# Supplementary figures and images for: CRACC-CRACC Interaction between Kupffer and NK Cells Contributes to Poly I:C/D-GalN Induced Hepatitis
Source: PLoS One. 2013 Sep 30;8(9):e76681. doi: 10.1371/journal.pone.0076681 (PMC3786926; doi:10.1371/journal.pone.0076681)

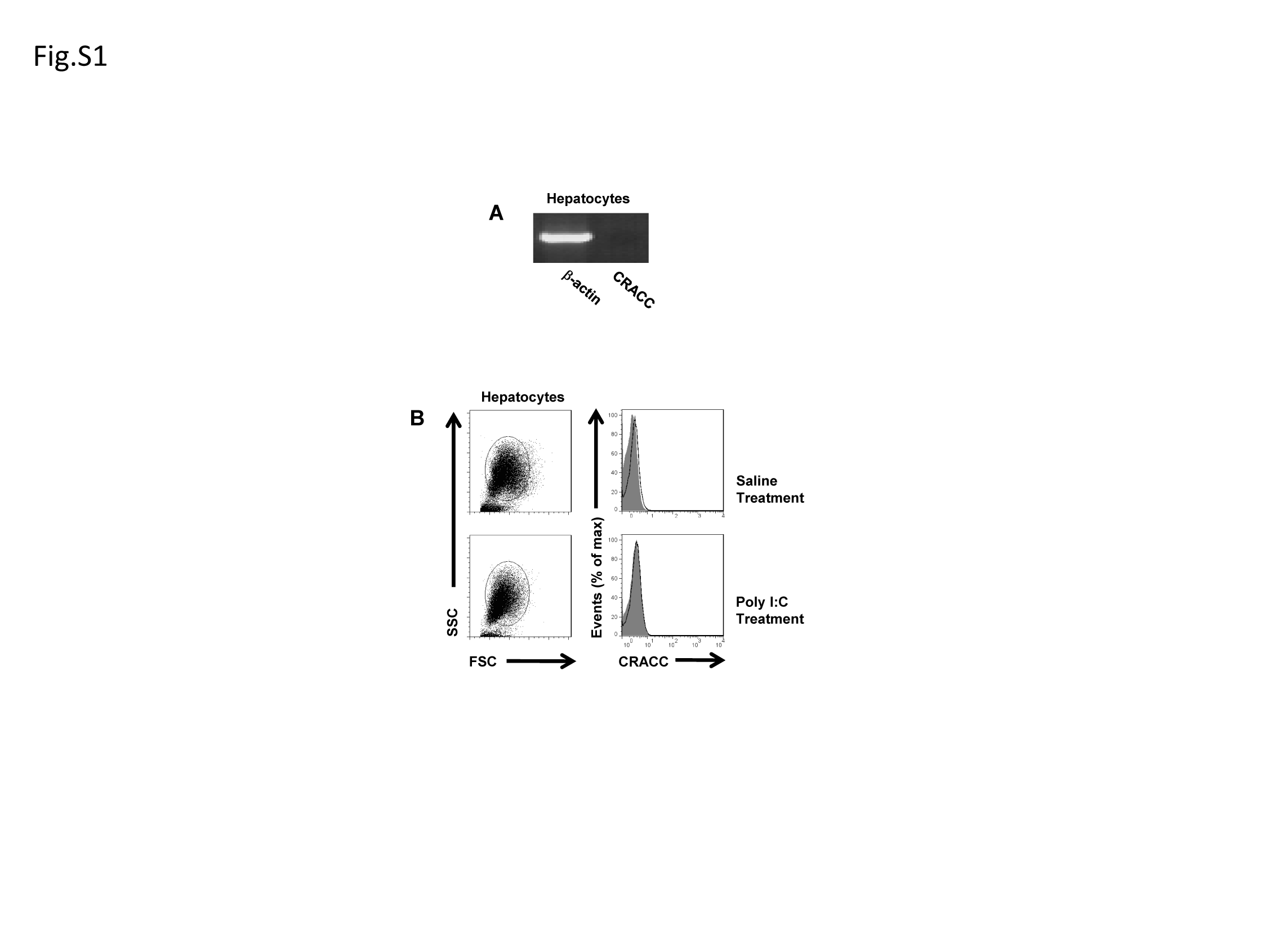

Supplement: Figure S1 — CRACC is undetectable on hepatocytes. A, CRACC mRNA expression of murine hepatocytes was assayed by RT-PCR. B, Mice were treated with Poly I:C, and CRACC expression on hepatocytes was analyzed by flow cytometry at 18h time point post the Poly I:C treatment. (TIF) [file pone.0076681.s001.tif]

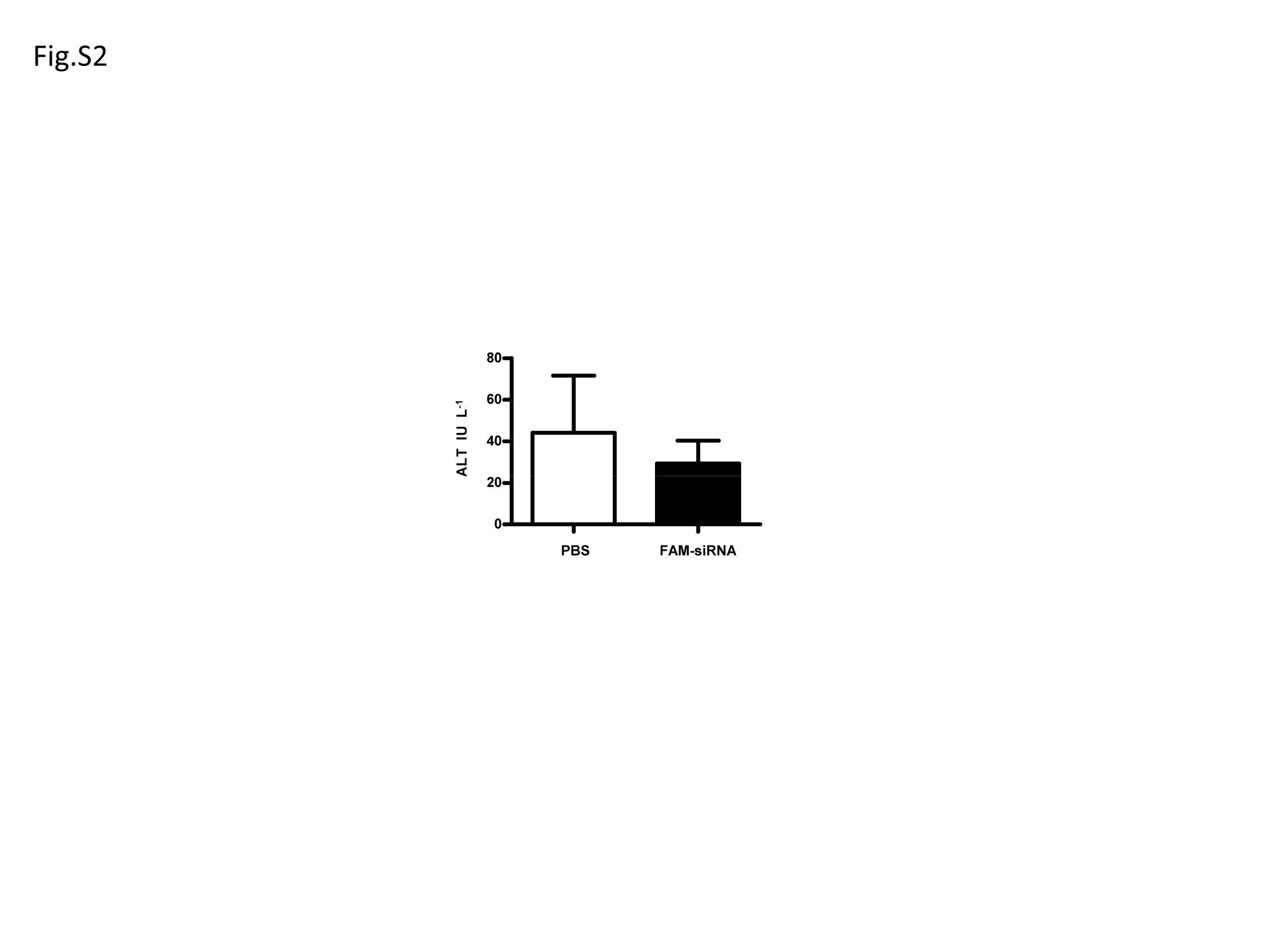

Supplement: Figure S2 — Nanoparticle encapsulated FAM conjunct siRNA induces no liver injury. Mice were treated with nanoparticle encapsulated FAM conjunct siRNA by intravenous injection; and the serum ALT was tested 3h later. (TIF) [file pone.0076681.s002.tif]

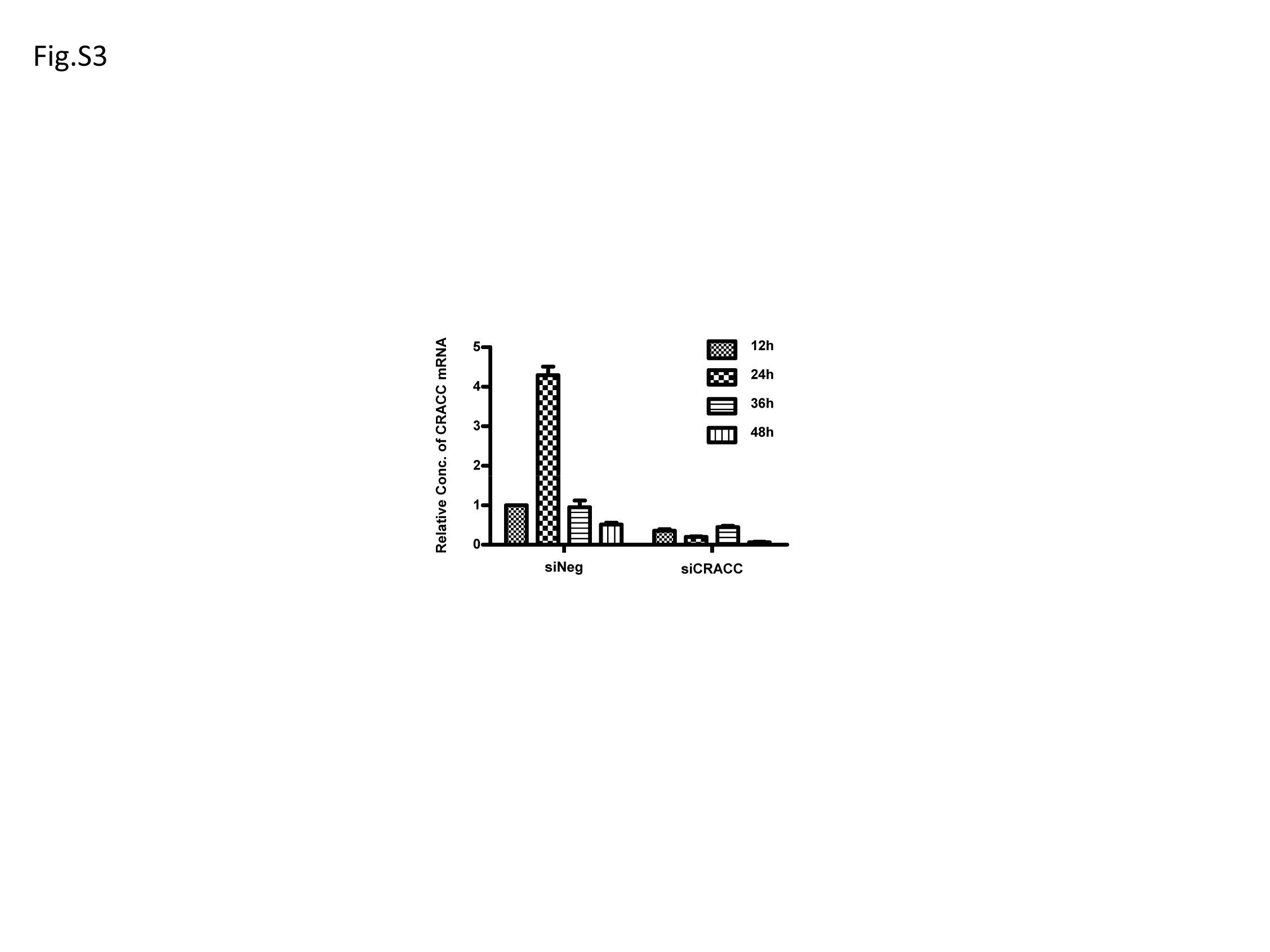

Supplement: Figure S3 — The CRACC expression is silenced by siCRACC. 293A cells were transfected with pcDNA3.0-CRACC together with siNeg or siCRACC. The expression of CRACC on 293A cells was assayed by quantitative PCR at 12h, 24h, 36h and 48h time points. (TIF) [file pone.0076681.s003.tif]
